# Effects of Hyperbranched Polyester-Modified Carbon Nanotubes on the Crystallization Kinetics of Polylactic Acid

**DOI:** 10.1021/acsomega.1c00738

**Published:** 2021-04-06

**Authors:** Fuyi Zhang, Weijiao Jiang, Xiuduo Song, Jian Kang, Ya Cao, Ming Xiang

**Affiliations:** † State Key Laboratory of Polymer Materials Engineering, Polymer Research Institute of Sichuan University, Chengdu 610065, China; ‡ Key Laboratory of Combustion and Explosion Technology, Xi’an Modern Chemistry Research Institute, Xi’an 710065, China

## Abstract

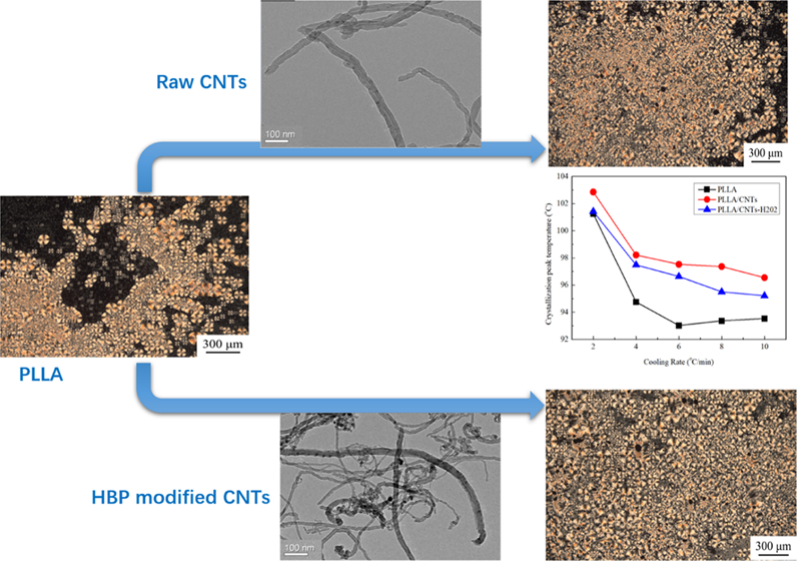

Poly-l-lactic acid (PLLA) is a prospective renewable and degradable material, but slow crystallization limits its processing and application. By dehydration condensation of hydroxyl-terminated hyperbranched resin (H202) and carboxylated carbon nanotubes (CNTs), a modified CNT, CNTs-H202, was obtained. Grafting was confirmed by Fourier transform infrared (FTIR) spectroscopy, and the grafting content was assessed by thermogravimetric analysis (TGA). Changes in surface atom content were explored by X-ray electron spectroscopy (XPS). Transmission electron microscopy (TEM) observed the increase of black dots on the surface of carbon nanotubes. PLLA/CNTs and PLLA/CNTs-H202 composites were prepared, and differential scanning calorimetry (DSC) was used to investigate the crystallization behavior of the composites. The results showed that during the cooling process, PLLA/CNTs-H202 had a larger crystalline full width at half-maximum (FWHM) compared with PLLA/CNTs and exhibited the ability to hinder chain segment movement during the subsequent reheating process. The crystallization activation energy was calculated by the Kissinger method, and it was found that the activation energy of the carbon tube increased slightly after grafting. Wide-angle X-ray diffraction (WAXD) once again proved the improvement of the crystallization ability. The results of polarized optical microscopy (PLOM) showed that the number of crystal nuclei increased and the crystal became smaller.

## 1 Introduction

Poly-l-lactic acid (PLLA), one of the most prospective renewable materials, has been widely used in environmentally friendly packaging,^1^ three-dimensional (3D)-printing materials,^2^ cloth manufacturing, and other fields for its outstanding properties such as proper melting point and relatively high strength, biocompatibility, biodegradability, and antibacterial properties.^3^ However, further utilization of PLLA has been limited because of its intrinsic defects such as slow crystallization rate and brittleness. Therefore, increasing the crystallization rate has become one of the focus points of the current study.^4,5^ Relatively high crystallinity provides greater strength and lower degradation rates.^6^ Improving crystallinity by adding nucleating agents has been intensively studied.^7−9^ Kawamoto et al.^10^ explored a series of nucleating agents containing hydrazide groups, and among all of the samples, benzoylhydrazide-containing and longer-methylene-chain nucleating agents achieved higher crystallization temperatures and crystallinity.

Carbon nanotubes (CNTs) have been attracting huge interest since their discovery. High aspect ratio and outstanding mechanical, electrical, and thermal properties make them a prospective modifier.^11,12^ However, some drawbacks such as high surface energy may limit the use of CNTs, making it difficult to be mixed homogeneously in the polymer matrix and eventually degrading the performance of composites. To solve this problem, surface modification is a typical solution. Luo et al.^13^ reported polycaprolactone-modified multiwalled carbon nanotubes as a nucleating agent and plasticizer in PLLA, and the results showed that the decomposition temperature of the composites increased by about 30 °C. Iwata et al.^3^ investigated the effects of ethyl acetate-modified multiwalled carbon nanotubes on the PLLA crystallization behavior, and the results indicated that the addition of functional nanotubes makes spherulites smaller and more homogeneous.

Hyperbranched polymers (HBPs) have more special properties than linear polymers due to their unique branched structure. This feature decreases intermolecular entanglement compared with linear macromolecules. As a result, many scholars have tried to improve the toughness of PLLA with HBPs:^14−16^ Zhang et al.^17^ blended PLLA with hyperbranched polymer and investigated the mechanical properties and crystallization behavior. The result suggested that the tensile strength and elongation at break were significantly improved as well as the crystallization rate was increased. At the same time, the abundant functional groups provide plenty of reactive sites, which makes HBPs desirable macromolecular modifiers in the reaction with CNTs. Nevertheless, the effect of HBP-modified CNTs on the crystallization behavior of PLLA has been rarely reported. In this study, the role of HBP surface modification of CNTs in the crystallization kinetics of PLLA was comparatively investigated and the impacts of CNTs and modified CNTs were comparatively discussed.

## 2 Experimental Section

### 2.1 Materials and Sample Preparation

#### 2.1.1 Materials

Poly-l-lactic acid (Ingeo 4032D grade) was purchased from NatureWorks Corp. The number-averaged molecular weight was 52 000 g/mol,^18^ and the density was 1.24 g/cm^3^. Carboxylated carbon nanotubes (M1-CH, CNTs) were obtained from BOYU GAOKE New Material Technology Co. Ltd., China. Hyperbranched polyester (HBP) with a terminal hydroxyl group (H202) was produced by Wuhan HyperBranched Polymers Science & Technology Corp. Ltd., China. Its molecular weight is 1200 g/mol, and the number of hydroxyl groups was 10–12 mol/mol. *N*,*N*-dimethylformamide (DMF) was purchased from Beijing Additives Institute, China, and was used as received. Tetrabutylammonium bromide (TBAB) served as a catalyst.

#### 2.1.2 Preparation of Modified CNTs

HBP H202-modified CNTs were prepared according to the following procedure: 1 g of CNTs, 1 g of TBAB, and 5 g of HBP H202 were put into a beaker, then 200 mL of DMF were added. After ultrasonic agitation for 2 h, a uniform suspension was obtained. Then, it was transferred into a round-bottom flask and kept at 120 °C in an oil bath for 24 h under agitation by a magnetic stirrer. The product was washed with acetone for a few times and then filtered with a poly(vinylidene difluoride) (PVDF) membrane (pore size, 0.22 μm). The washing step was repeated using deionized water to ensure that all unreacted HBP H202 was removed. Finally, the product was placed in a plate and dried at 60 °C for 12 h in a vacuum oven for further application.

#### 2.1.3 Preparation of CNTs/PLLA Composites

The PLLA/CNTs or PLLA/CNTs-H202 mixture (1:0.005 weight ratio) was added into an RM-200C torque rheometer as a blender under a temperature of 200 °C and kept for 5 min to ensure proper plasticity. Then, the mixture was stirred for about 10 min at a speed of 50 rpm under a temperature of 200 °C. To prevent possible degradation during the processing process, a 0.1 wt % phenolic antioxidant agent (Irganox 1010, Beijing Additives Institute, China) was added. For further measurements, thin-sheet samples were prepared. The samples were first molded at 200 °C and 10 MPa for 6 min into thin sheets of 1 mm thickness. Then, cold pressing for 4 min was applied.

### 2.2 Characterization

#### 2.2.1 Fourier Transform Infrared (FTIR) Spectroscopy

A Nicolet 560 (Nicolet Corp.) Fourier transform infrared spectrometer with wavenumber from 400 to 4000 cm^–1^ and resolution of 4 cm^–1^ was used.

#### 2.2.2 X-ray Photoelectron Spectroscopy (XPS)

An Axis Ultra DLD X-ray photoelectron spectrometer (Kratos, U.K.) was used to characterize C, N, and O atom contents. The analyzer scanning mode was CAE, the X-ray source was mono Alka, energy was 1486.6 eV, voltage was 15 kV, beam current was 15 mA, and instrument work function was 4.2. The relative content of carbon and oxygen atoms was calculated using the following formula
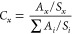
1where *C_x_* is the concentration of the desired element, _*Ax*_ is its peak area, *S*_*x*_ is its sensitivity factor, and ∑*A*_*i*_/*S*_*i*_ is the summation of the ratios of all measured peak areas to the sensitivity factor, where the sensitivity factors of C 1s and O 1s are 0.278 and 0.780, respectively.

#### 2.2.3 Transmission Electron Microscopy (TEM)

The morphology characterization was carried out by a Tecnai G^2^ F20 (FEI) with an acceleration voltage of 200 kV and a two-point resolution of 0.24 nm.

#### 2.2.4 Thermogravimetric Analysis (TGA)

Thermogravimetry was used to analyze the content of grafted HPB H202. It was conducted using a TG209F1 thermogravimetric analyzer (Netzsch Corp., Germany) with a heating rate of 10 °C/min, from 30 to 800 °C, under a nitrogen atmosphere.

#### 2.2.5 Differential Scanning Calorimetry (DSC)

All of the calorimetric experiments were performed with a Mettler Toledo DSC1 differential scanning calorimeter, which was calibrated by indium under a nitrogen atmosphere at the rate of 50 mL/min to ensure the reliability of the obtained data. To ensure the homogeneity of samples and good thermal conductivity between samples and pans, the composite was molded at 190 °C and 10 MPa for 10 min into sheets with a uniform thickness of about 1 mm. Then, round samples (5 mg) were cut from the sheets. The experimental error of the measured temperature was ±0.1 °C. The crystallinity *X*_*c*_ of PLLA and its composites was calculated according to the following equation
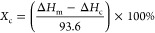
2where Δ*H*_c_ is the enthalpy of crystallization during PLLA cooling, Δ*H*_m_ is the enthalpy of fusion, and 93.6 J/g is the melting enthalpy of PLLA with infinite crystal thickness.^19^

For the purpose of investigating nonisothermal crystallization kinetics, the procedures were set as follows: the samples were first heated to 200 °C for 8 min to eliminate thermal history. Then, they were cooled to 30 °C at various cooling rates of 2, 4, 6, 8, and 10 °C/min. A subsequent heating at the rate of 10 °C/min to 200 °C was carried out to characterize its melting behavior.

#### 2.2.6 Wide-Angle X-ray Diffraction (WAXD)

An Ultima IV X-ray diffractometer (RIGAKU Corp., Japan) was used to perform WAXD test on the composites with Cu Kα rays as the radiation source, 40 kV voltage, 100 mA current, 2θ = 5–60°, and λ = 1.54 Å.

#### 2.2.7 Polarized Optical Microscopy (PLOM)

The crystal morphology of PLLA and its composites was observed by a Nikon ECLIPSE LV100N POL polarizing microscope (Nikon Corp., Japan).

## 3 Results and Discussion

### 3.1 Characterization of CNTs and CNTs-H202

#### 3.1.1 FTIR Analysis

**Figure 1:**
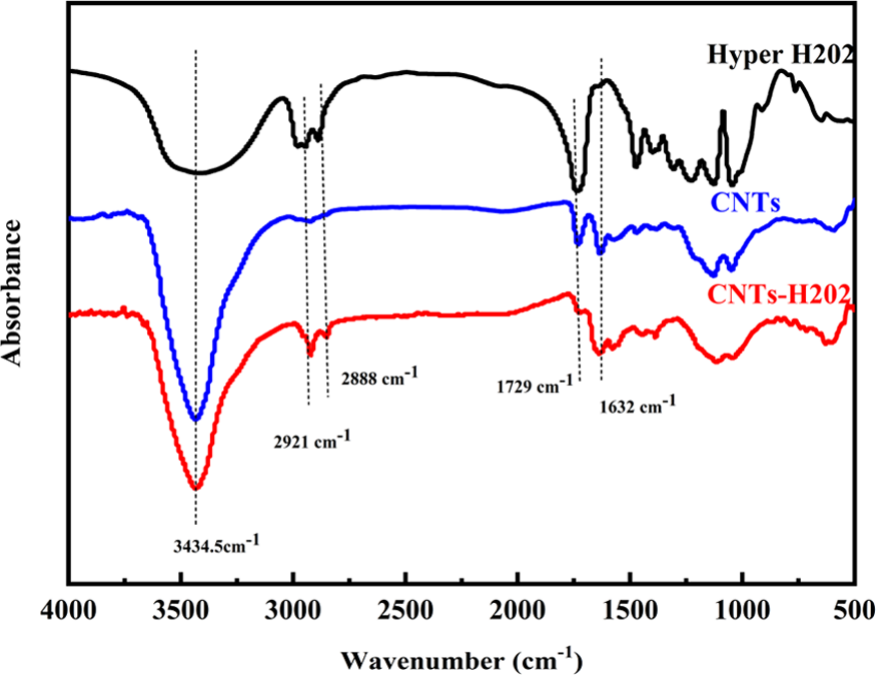
FTIR spectra of CNTs, CNTs-H202, and H202.

The FTIR spectra of CNTs, CNTs-H202, and HBP H202 are shown in Figure 1. For HBP H202, the wide absorption peak at 3434 cm^–1^ is the stretching vibration peak of hydroxyl groups in hyperbranched polyester. The peaks at wavenumbers of 2921 and 2888 cm^–1^ indicate the antisymmetric and symmetric stretching vibrations of methyl and methylene groups, respectively. The peak at 1729 cm^–1^ represents the stretching vibration of the carbonyl group.^20^ In the spectrum of CNTs and CNTs-H202, the peak at 1574 cm^–1^ is the typical C=C stretching vibration absorption peak. Compared with CNTs, CNTs-H202 has stronger antisymmetric and symmetric vibrations of methyl and methylene groups at 2921 and 2888 cm^–1^, respectively, suggesting that H202 has been successfully grafted on the surface of CNTs. To obtain more accurate results, X-ray photoelectron spectrometry (XPS) was also used for further characterization.

#### 3.1.2 X-ray Photoelectron Spectrometry (XPS)

**Figure 2:**
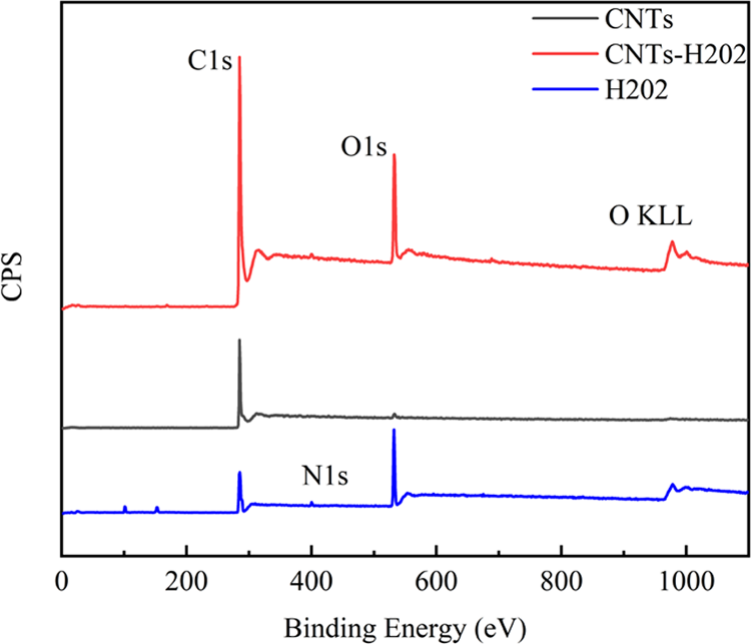
XPS survey spectra of CNTs, CNTs-H202, and HBP H202.

To confirm that H202 has been grafted onto carbon nanotubes, XPS was performed on CNTs, CNTs-H202, and H202. The XPS results are demonstrated in Figure 2. The percentages of O 1s and C 1s as well as the O/C ratio (*n*_O_/*n*_C_) were calculated and are listed in Table 1.

**Table 1:** Contents of Surface Composition of CNTs, CNTs-H202, and HBP H202

sample	C 1s (%)	O 1s (%)	*n*_O_/*n*_C_
CNTs	97.39	2.61	0.027
CNTs-H202	86.92	13.08	0.15
HBP H202	31.78	68.22	2.15

The 2.61% O atoms in raw CNTs mainly come from the acidization process, during which many carboxy groups as well as a large number of hydroxyl groups were introduced onto the surface of CNTs, which was proved by previous works.^21−23^ From Figure 2, CNTs-H202 shows a lower C 1s intensity and a higher O 1s intensity compared with CNTs and exhibits a significant increase in O/C ratio (0.15) compared with raw CNTs, owing to the contribution of HBP H202 with the highest O/C ratio of 2.15.

#### 3.1.3 TGA Measurement

**Figure 3:**
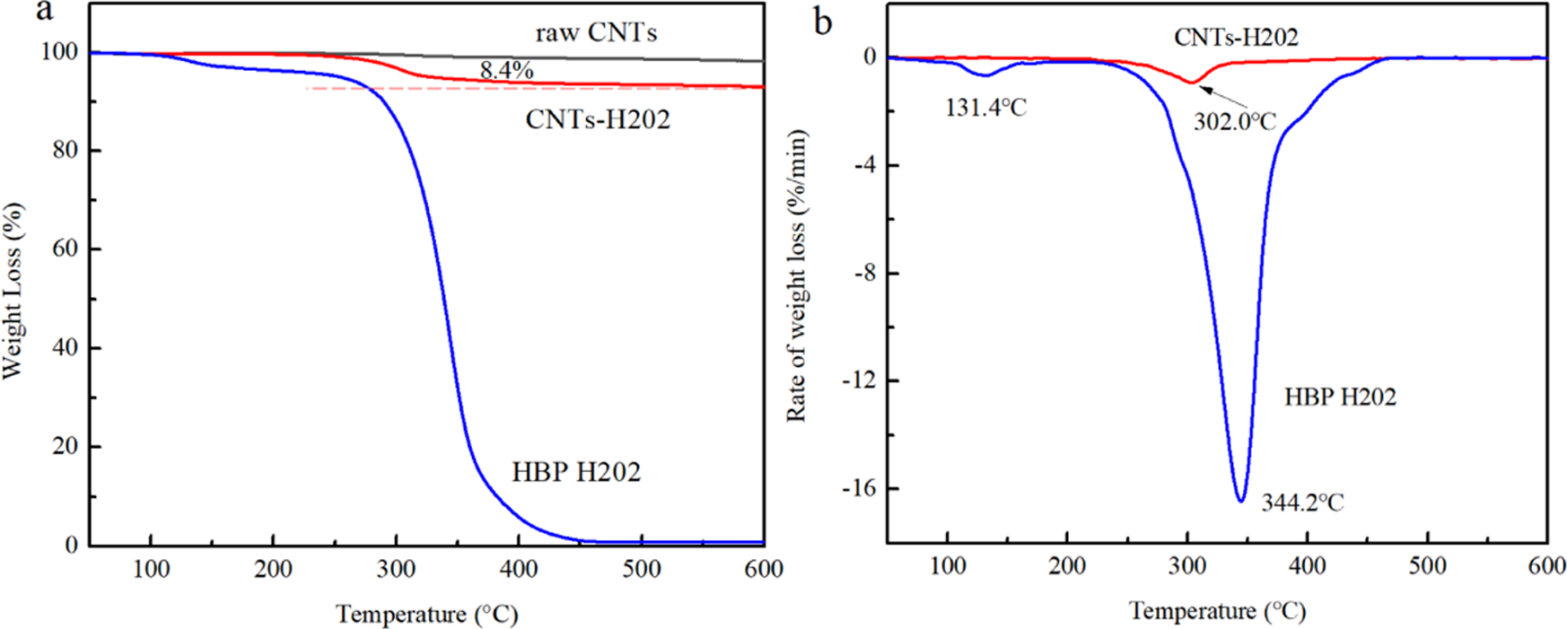
(a) TGA plot of CNTs, CNTs-H202, and H202. (b) DTG plot of CNTs-H202 and HBP H202.

TGA was performed on HBP H202, CNTs, and CNTs-H202, and the results are shown in Figure 3. Figure 3a shows thermogravimetry curves, while Figure 3b shows DTG plots of the samples. It can be observed from Figure 3a that the weight loss of HBP H202 mainly occurs in two steps, and the maximum weight loss temperatures are 131.4 and 344.2 °C, respectively. For raw CNTs, no obvious weight loss can be observed in the temperature range of 50–600 °C, due to its high thermal stability.^24^ Therefore, the residual content at 600 °C can be considered as 100%. For CNTs-H202, the maximum weight loss temperature is 302 °C, suggesting that HBP H202 has been grafted onto CNTs. Moreover, an 8.4% weight loss can be seen at the temperature of 450 °C. Compared with HBP H202, it can be roughly estimated that the grafting amount of H202 is 8.4%.

#### 3.1.4 TEM Observation

**Figure 4:**
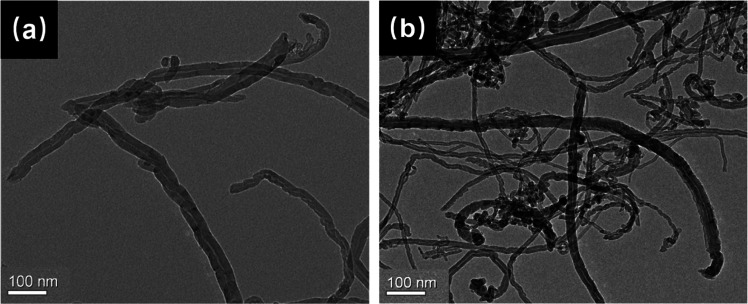
TEM images of (a) raw CNTs and (b) CNTs-H202.

To directly observe the morphologies of raw CNTs and CNTs-H202, TEM measurement was performed. The TEM images of raw CNTs and CNTs-H202 are shown in Figure 4. For raw CNTs (Figure 4a), typical morphology of CNTs can be clearly observed. Interestingly, for CNTs-H202 in Figure 4b, many relatively dark dots can be observed, which might be the grafted HBP H202. Generally, it can be concluded that after the HBP H202 surface modification, the surface morphology of the CNTs has been significantly changed.

### 3.2 Crystallization and Melting Behavior of PLLA/CNTs Composites

**Figure 5:**
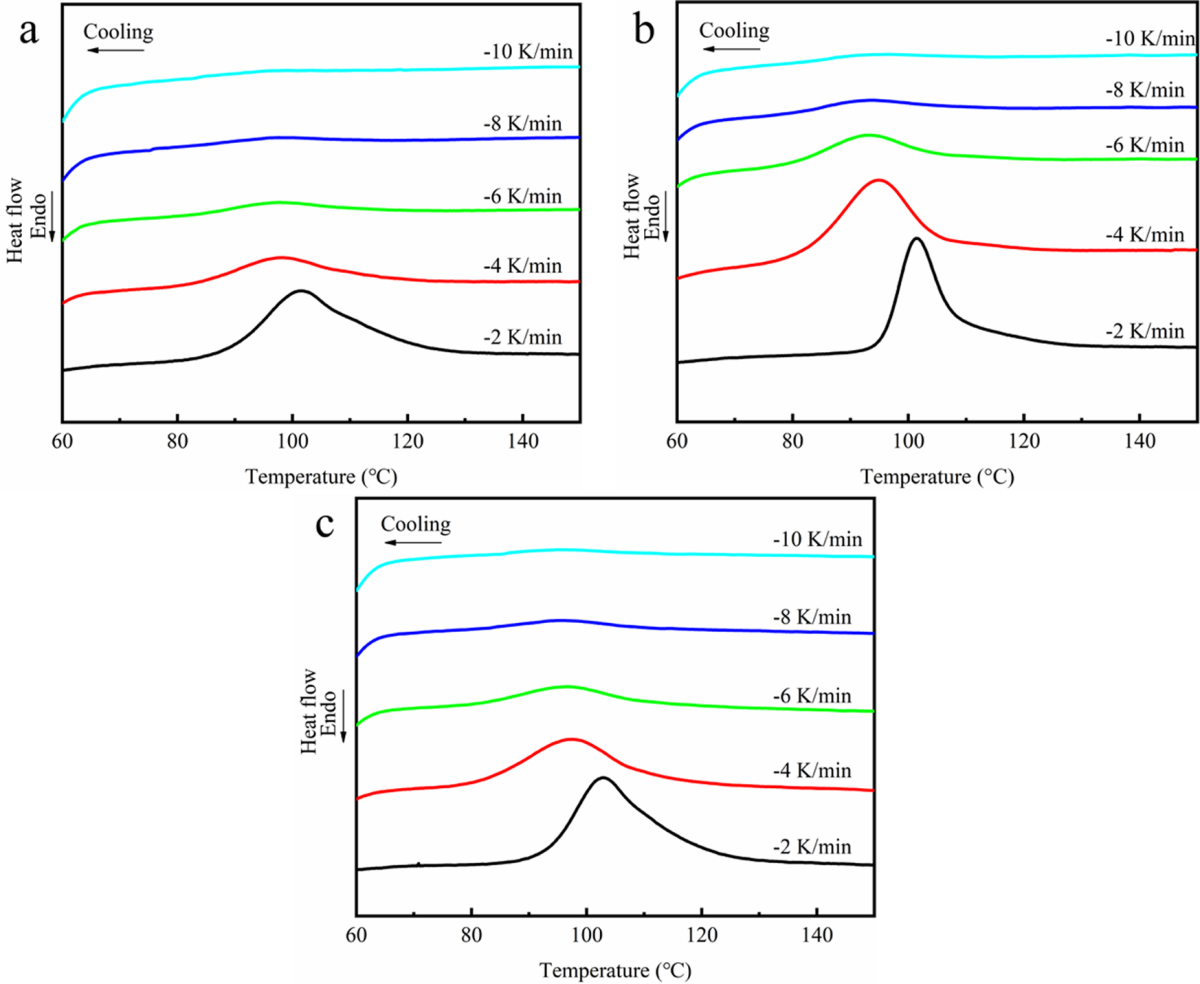
Cooling curves of PLLA (a), PLLA/CNTs (b), and PLLA/CNTs-H202 (c) at different cooling rates.

**Figure 6:**
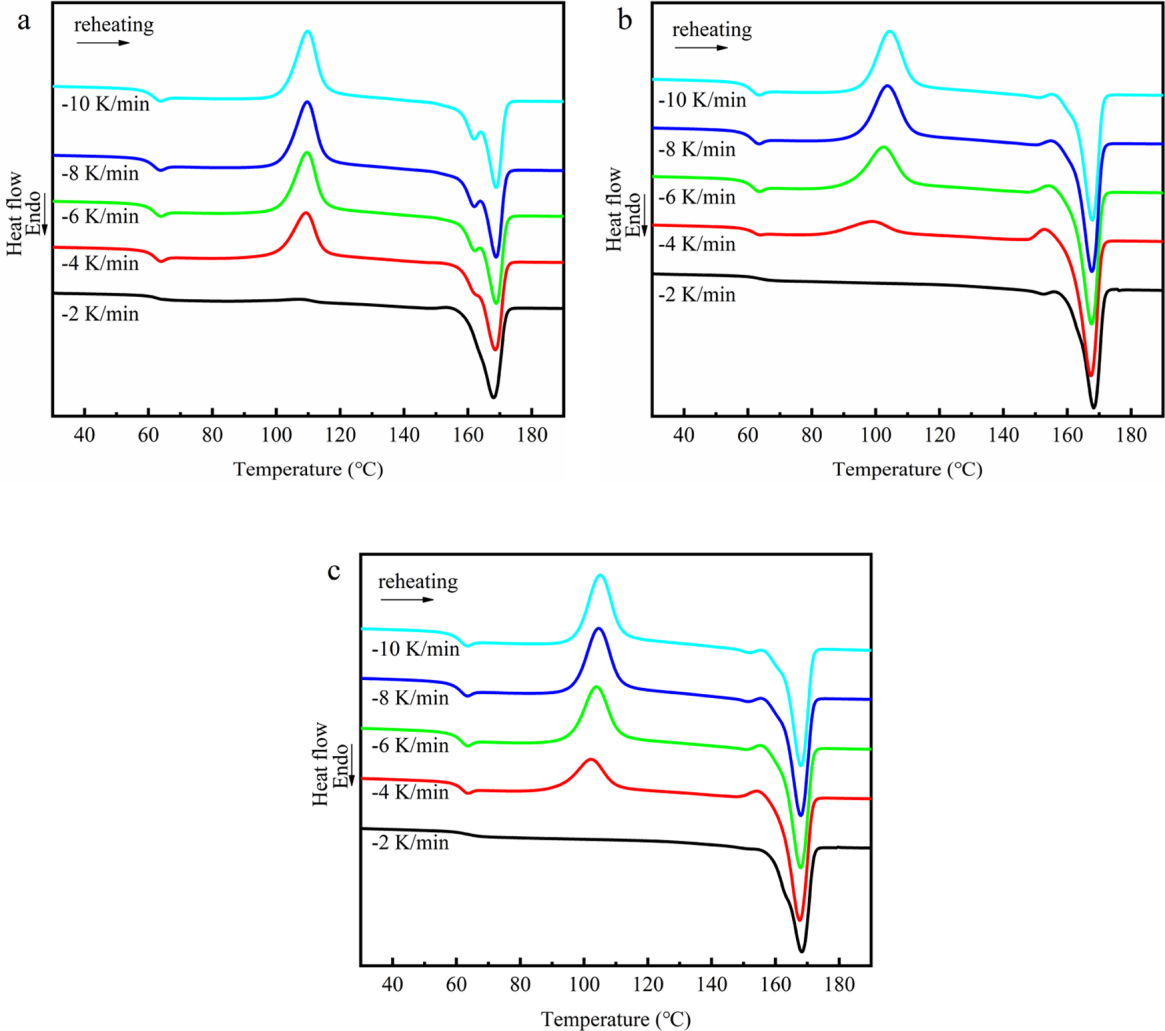
Melting curves at the heating rate of 10 °C after cooling at different rates of (a) PLLA, (b)PLLA/CNTs, and (c) PLLA/CNTs-H202.

**Figure 7:**
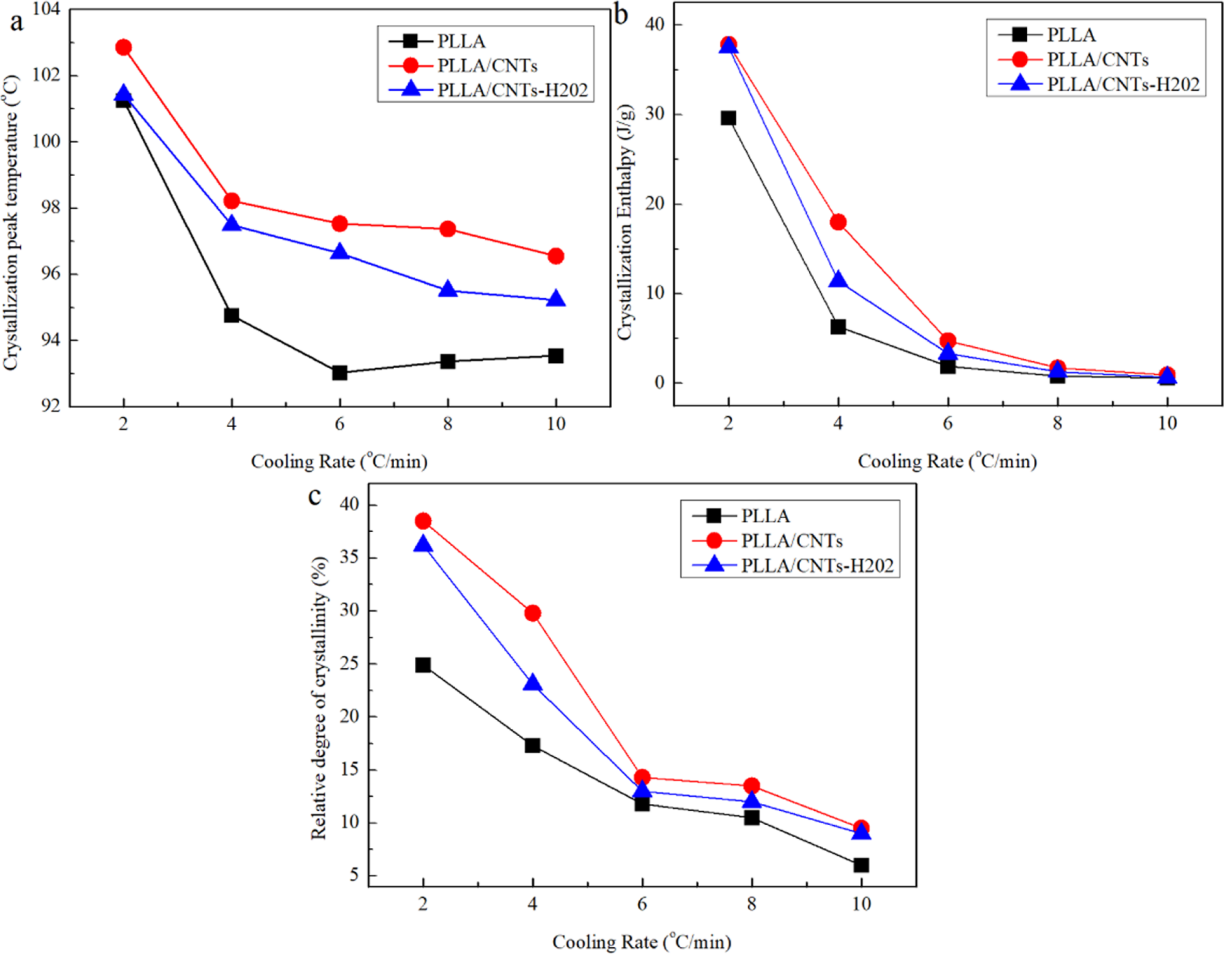
Crystallization and melting parameters of PLLA, PLLA/CNTs, and PLLA/CNTs-H202. (a) Crystallization peak temperature *T*_c_, (b) crystallization enthalpy, and (c) relative degree of crystallinity.

To comparatively study the crystallization and melting behavior of PLLA/CNTs and PLLA/CNTs-H202, samples were first heated to 200 °C for 8 min to eliminate thermal history and then cooled to 25 °C at various cooling rates of 2, 4, 6, 8, and 10 °C/min, respectively. A subsequent reheating at the rate of 10 °C/min to 200 °C was carried out to characterize its melting behavior. The cooling curves of PLLA, PLLA/CNTs, and PLLA/CNTs-H202 at different cooling rates are plotted in Figure 5, and the subsequent heating scans at the rate of 10 °C/min are shown in Figure 6. From Figures 5 and 6, the crystallization and melting parameters, including crystallization peak temperature *T*_c_, crystallization enthalpy Δ*H*_cc_, and relative degree of crystallinity *X*_c_, are shown in Figure 7.

It can be seen from Figure 5 that for all samples, the slower the cooling rate is, the more obvious the signal of cooling crystallization peak is, indicating that the crystallization rates of PLLA and its composites are slow and their crystallization behaviors are strongly dependent on the cooling rate. At the same cooling rate, the crystallization curve of pure PLLA is the weakest. After adding CNTs or CNTs-H202, the crystallization peak becomes more obvious; when the cooling rate is 2 °C/min, the width of the crystallization peak of the composites becomes narrower, which indicates that CNTs or CNTs-H202 can promote the crystallization of PLLA.

The melting curve of Figure 6 further proves the crystallization promotion effect of CNTs. On the melting curve, the glass-transition temperature (about 65 °C), the cold crystallization peak (between 90 and 130 °C), and the melting peak (above 150 °C) of PLLA can be observed. It can be seen that for all samples, there is almost no cold crystallization peak at the slow cooling rate of 2 °C/min, which might be explained as follows: the slow cooling rate makes the crystallization more sufficient in the cooling process; therefore, the cold crystallization peak is almost invisible in the subsequent heating and melting process. In other words, when the cooling rate is the same, the weaker the cold crystallization peak signal, the stronger the crystallization ability of PLLA. Comparing different samples, it can be seen that when the cooling rate is −4 °C/min, the cold crystallization peak signals of PLLA/CNTs and PLLA/CNTs-H202 are lower than that of pure PLLA, and that of PLLA/CNTs is the lowest, indicating that CNTs have the largest effect on the crystallization of PLLA, followed by CNTs-H202.

Figure 7 shows the crystallization and melting behavior parameters obtained in Figures 5 and 6. It can be seen that pure PLLA has the lowest crystallization peak temperature *T*_c_ at all cooling rates, which indicates that its crystallization ability is the weakest; after the addition of CNTs or CNTs-H202, *T*_c_ of the composite increases significantly, which indicates that both of them can promote the crystallization of PLLA. Compared with PLLA/CNTs-H202, PLLA/CNTs have higher *T*_c_’s, indicating that the PLLA/CNTs have a more significant effect on promoting crystallization. Moreover, the results of crystallization enthalpy and relative crystallinity are consistent.

### 3.3 Crystallization Activation Energy

For the crystallization process, activation energy (Δ*E*) is a vital parameter, which represents the energy barrier for the polymer to migrate from the melt to the crystal surface. The larger the value of Δ*E* is, the more difficult the melt crystallizes.^25,26^ Kissinger proposed a method to evaluate the activation energy formula of the crystallization process as follows
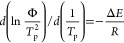
3

where Φ is the heating or cooling rate, *T*_p_ is the crystallization peak temperature, Δ*E* is the activation energy of crystal diffusion, and *R* is the gas constant. The calculated activation energies of the samples via the Kissinger method are listed in Table 2.

**Table 2:** Activation Energies of the Samples Derived from the Kissinger Method

sample	*E* (kJ·mol^–1^)
PLLA	–106.77
PLLA/CNTs	–134.33
PLLA/CNTs-H202	–124.02

Obviously, PLLA/CNTs have the lowest activation energy, which means it is the easiest to crystallize. The largest value was PLLA. It can be seen that, no matter whether the carbon nanotubes are modified or not, they can effectively reduce the crystallization activation energy and make the crystallization process easier. PLLA/CNTs-H202 requires a higher activation energy than PLLA/CNTs, which might be explained as follows: compared with raw CNTs, CNTs-H202 has greater steric hindrance, which might increase the activation energy of crystallization and make the crystallization more difficult.

### 3.4 WAXD Measurement

**Figure 8:**
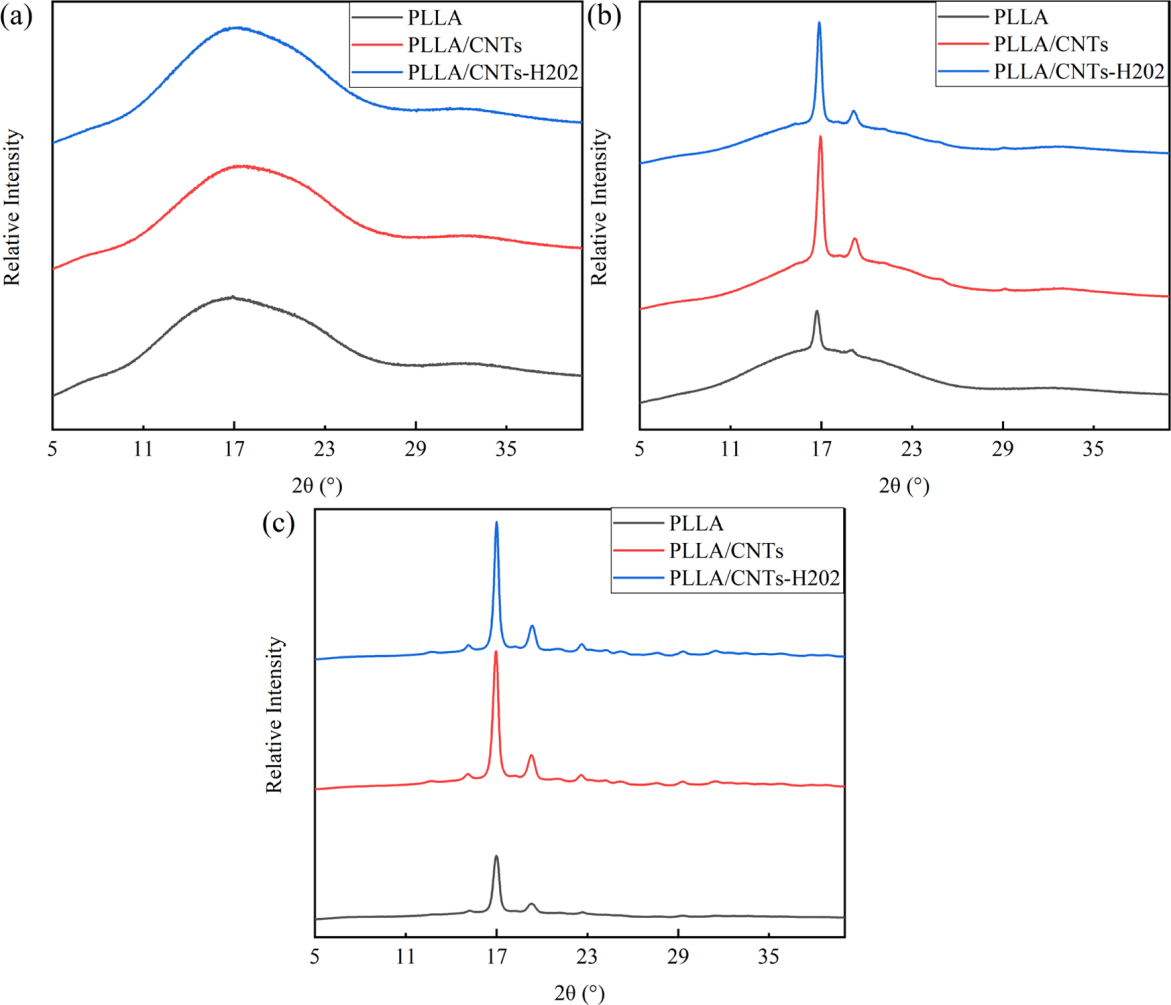
WAXD spectrum of PLLA and its composites at the cooling strategies of (a) fast cooling, (b) medium cooling, and (c) slow cooling.

To investigate the impact of CNTs and modified CNTs on the crystallization of PLLA thoroughly, samples with various cooling rates were prepared for WAXD measurement as follows: to obtain a relatively large specimen for the WAXD measurement, in this section, we prepared the specimens using the heating oven. The samples were first placed in a heating oven at 200 °C for 10 min to eliminate thermal history. Then, heating of the oven was stopped and the specimens were cooled by different strategies: For fast cooling specimen, it was taken out of the oven and rapidly cooled at room temperature; the estimated cooling rate was 200 °C/min. For medium cooling specimen, only the door of the oven was half-opened, and it was cooled to room temperature in about 20 min; therefore, the estimated cooling rate was 8–10 °C/min. For slow cooling specimen, it was slowly cooled at the oven with the door closed. The estimated cooling rate was 1 °C/min. The obtained WAXD profiles are shown in Figure 8.

As can be seen from Figure 8a, there was only amorphous PLLA during the rapid cooling process. The reason is that it is difficult for PLLA segments to arrange into the lattice under such an insufficient crystallization condition. The addition of CNTs and CNTs-H202 increases the number of crystal nuclei but has little influence on promoting the movement of the segments of PLLA. In Figure 8b, a distinct peak at 2θ = 16.7° corresponding to the characteristic diffraction of (110)/(200) plane can be observed, while another obvious peak locates at 2θ = 19.1° corresponding to the characteristic diffraction of the (203) plane^27^ can be seen. The intensities of these peaks of PLLA-CNTs are the highest among the three samples, while those of pure PLLA are the lowest, suggesting that under medium cooling rate, the crystallinity of PLLA/CNTs is the highest. In other words, raw CNTs had the highest effect for accelerating crystallization of PLLA. When the cooling rate is very low (slow cooling samples), the intensities of these peaks become much higher than their counterparts cooled at a medium rate. The peaks of PLLA/CNTs and PLLA/CNTs-H202 are sharper than those of pure PLLA, indicating that the presence of CNTs or CNTs-H202 accelerates the crystallization of PLLA.

### 3.5 PLOM Observation

**Figure 9:**
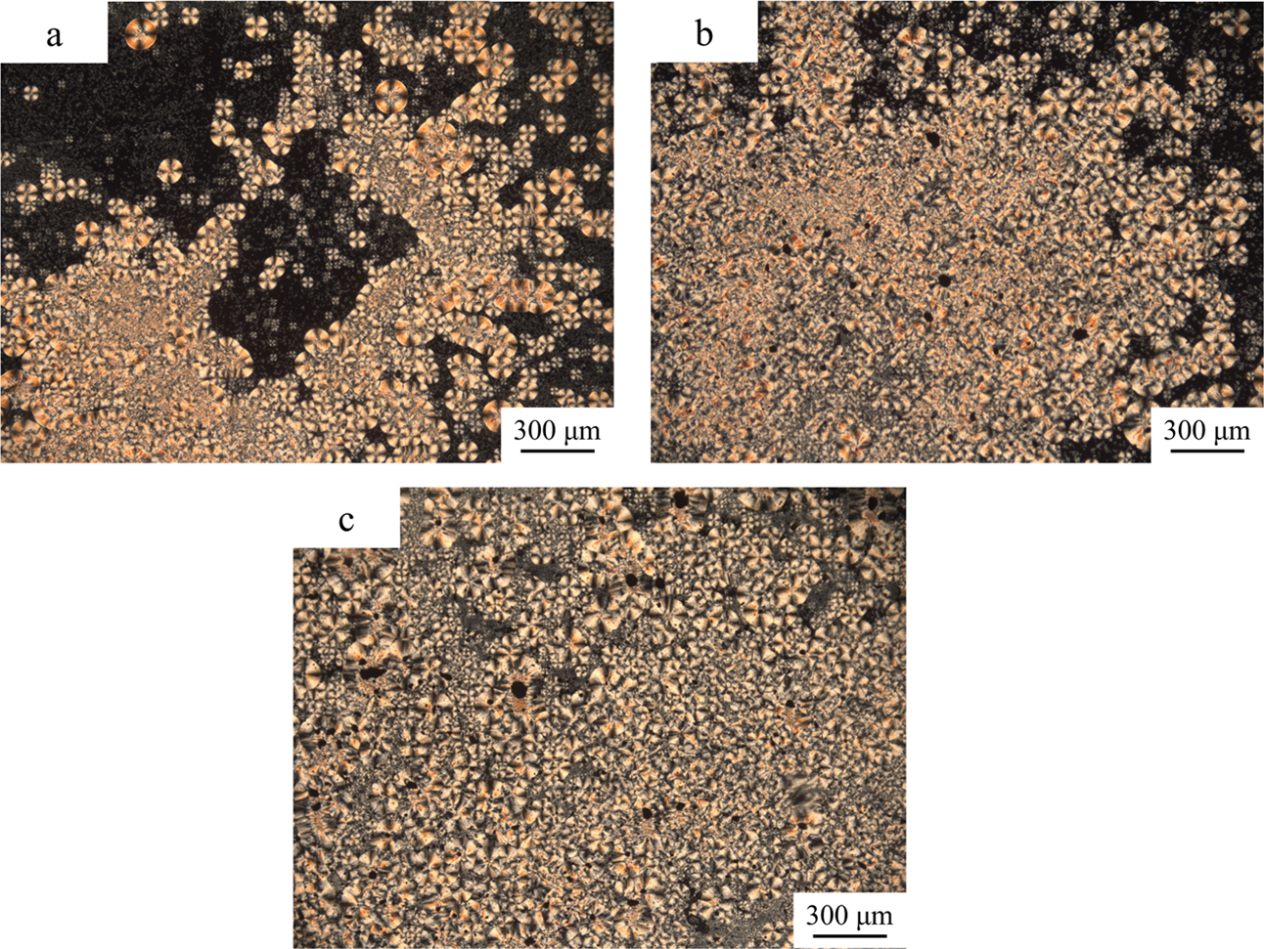
PLOM images of the samples after isothermal crystallization: (a) PLLA, (b) PLLA/CNTs, and (c) PLLA/CNTs-H202.

The samples were heated to 195 °C for 5 min to eliminate thermal history and then cooled to 120 °C for isothermal crystallization for 30 min. The obtained PLOM images are shown in Figure 9.

Figure 9 shows the crystalline morphologies of the samples after isothermal crystallization. For pure PLLA, after isothermal crystallization for 0.5 h, many spherulites can be seen. However, about half of the image is dark, suggesting that the crystallization has not finished. From Figure 9b,c, there were crystals throughout the field of view; thus, it was evident that the crystallinity of PLLA/CNTs and PLLA/CNTs-H202 was higher than that of pure PLLA. In addition, in terms of spherulite size, pure PLLA showed larger spherulite due to the slow crystal growth rate, while PLLA/CNTs and PLLA/CNTs-H202 showed smaller and more overlapped crystals due to the addition of CNTs or CNTs-H202 as a nucleating agent.

## 4 Conclusions

In this work, the HBP-modified CNTs were prepared by the condensation reaction. The grafting process was confirmed by means of XPS, FTIR, TGA, and TEM. PLLA/CNTs and PLLA/CNTs-H202 composites were prepared, and their crystallization and melting kinetics were comparatively investigated. The results showed that during the cooling process, PLLA/CNTs-H202 had a larger crystalline full width at half-maximum (FWHM) compared with PLLA/CNTs and exhibited the ability to hinder chain segment movement during the subsequent reheating process. The crystallization activation energy was calculated by the Kissinger method, and it was found that the activation energy of PLLA/CNTs-H202 increased slightly after grafting. WAXD measurement once again proved the improvement of the crystallization ability. The results of PLOM showed that the number of crystal nuclei increased and the crystal became smaller.
